# Study on Quality Standard of Processed* Curcuma Longa Radix*

**DOI:** 10.1155/2017/2830754

**Published:** 2017-12-11

**Authors:** Zhimin Chen, Yongfeng Zhao, Liang Quan, Haiting Zhou, Dong Cao, Changjiang Hu, Wenbing Li, Zhuo Yang

**Affiliations:** ^1^Chengdu University of TCM, Chengdu 611137, China; ^2^Chengdu Institution of Chinese Herbal Medicine, Chengdu 610016, China; ^3^Key Laboratory of Chinese Medicine Formulations Particle Mass and Clinical Evaluation, Chengdu 611900, China; ^4^Neo-Green Pharmaceutical Co., Ltd., Chengdu 611900, China

## Abstract

To control the quality of* Curcuma Longa Radix *by establishing quality standards, this paper increased the contents of extract and volatile oil determination. Meanwhile, the curcumin was selected as the internal marker, and the relative correlation factors (RCFs) of demethoxycurcumin and bisdemethoxycurcumin were established by high performance liquid chromatography (HPLC). The contents of multicomponents were calculated based on their RCFs. The rationality and feasibility of the methods were evaluated by comparison of the quantitative results between external standard method (ESM) and quantitative analysis of multicomponents by single-marker (QAMS). Ethanol extracts ranged from 9.749 to 15.644% and the mean value was 13.473%. The volatile oil ranged from 0.45 to 0.90 mL/100 g and the mean value was 0.66 mL/100 g. This method was accurate and feasible and could provide a reference for further comprehensive and effective control of the quality standard of* Curcuma Longa Radix* and its processed products.

## 1. Introduction

*Curcuma Radix* (Yujin) is derived from the dried root of* Curcuma wenyujin, Curcuma longa*,* Curcuma kwangsiensis,* or* Curcuma phaeocaulis*, which first appeared in the Theory of Drug Properties* (Yao Xinglun)* and has the effects of invigorating the circulation of blood, relieving pain, dispersing stagnated qi for relieving qi stagnation, clearing heat of heart and cooling blood, and curing jaundice [1–3].* Curcuma Longa Radix* (Huangsiyujin, HSYJ), as one of the main varieties of* Curcuma Radix*, is the famous genuine medicinal herbs produced in Sichuan. The* Tu Jing Ben Cao *which was written by Su Song recorded that “Guangnan and Jiangxi also have, but not as good as Sichuan” [4].

Modern pharmacological studies have shown that* Curcuma Longa Radix* has many functions, such as anti-inflammatory [5–7], easing pain [8], antithrombosis and platelet aggregation [9], antioxidation [10–12], antidepression, and cholagogic [13]. But the quality standard of* Curcuma Radix* is insufficient. In the Chinese Pharmacopoeia 2015 edition, only moisture and total ash are used as indicators for quality control of* Curcuma Radix*. It is difficult to fully reflect and control the quality of* Curcuma Longa Radix* due to the lack of quality control indicators. To date, HPLC has applied in determination of curcuminoids in* Curcuma Longa Radix* simultaneously [14]. However, due to high experimental cost, the application of this method was limited. Therefore, there is a clear need for the development of a quality control method. In this paper, in order to explore a comprehensive quality control criterion for* Curcuma Longa Radix *and guarantee the effective and safe use of clinical practice. We not only added the contents of extract and volatile oil determination but also developed and validated a quantitative analysis of multicomponents by single-marker (QAMS) for the simultaneous determination of polar active components in* Curcuma Longa Radix*, including curcumin, demethoxycurcumin, and bisdemethoxycurcumin.

## 2. Materials and Methods

### 2.1. Chemicals and Reagents

The reference standards of curcumin, demethoxycurcumin, and bisdemethoxycurcumin (purity ≥ 98%) were purchased from the National Institutes for Food and Drug Control (Beijing, China). Methanol and acetonitrile (Fisher, USA) were of HPLC grade. Other reagents were of analytical purity. Water was glass-distilled and filtered through a Milli-Q water purification system (Millipore, Bedford, MA) prior to use.* Curcuma Longa Radix* was gathered from geoauthentic habitats, such as Qianwei, Shuangliu, and Chongzhou in Sichuan province and processed in our own laboratory. We also purchased* Curcuma Longa Radix* from different TCM enterprises. These samples were authenticated by Professor Xianming Lu and Professor Guihua Jiang (Chengdu University of Traditional Chinese Medicine, Chengdu, China). The information of samples is shown in Table 1.

### 2.2. Determination of Ethanol Extracts

The ethanol extracts were determined according to the 2201 of Chinese Pharmacopoeia 2015 edition Volume IV. Homeopathic alcohol was used as the extraction solvent. The results were shown in Table 2.

### 2.3. Determination of Volatile Oil

The volatile oil was determined according to the 2204 of Chinese Pharmacopoeia 2015 edition Volume IV. The results were shown in Table 2.

### 2.4. Instrumentation and Separation Conditions

HPLC determinations were performed using an Agilent HPLC 1200 instrument (Agilent Technologies, Palo Alto, CA), equipped with a diode array detector (DAD) detector, an auto sampler, a column heater, and a Welch Ultimate® XB-C18 (250 mm × 4.6 mm, 5 *μ*m) column. The mobile phase consisted of A (acetonitrile) and B (4% glacial acetic acid aqueous) (V/V). Optimum separation was 48% A. The flow rate was 1.0 mL·min^−1^ and injection volume was 10 *μ*L. The column temperature was set at 30°C and the wavelengths were monitored at 425 nm.

### 2.5. Sample Preparation

Powder of* Curcuma Longa Radix* was precisely weighed (1 g) and immersed in 25 mL of methanol. Additional methanol was added to make up the loss after ultrasonic extraction for 30 min. For HPLC analysis, the filtrate was filtered through a filter (0.45 *μ*m pore size) prior to injection. And the negative control groups were prepared in the same manner.

### 2.6. Preparation of Standard Solution

A mixed stock solution containing reference standards was prepared by dissolving weighed accurately samples of each compound in methanol, which obtained curcumin 138.51 *μ*g·mL^−1^, demethoxycurcumin 28.20 *μ*g·mL^−1^, and bisdemethoxycurcumin 19.58 *μ*g·mL^−1^. The calibration curves were established by further dilution with methanol gives at least six different concentrations, and the six different concentrations of mixed standard solutions were injected (10 *μ*L) and calculated. The series of working solutions are within the ranges of 3.3242 to 13.851 *μ*g·mL^−1^ for curcumin, 0.6768 to 2.82 *μ*g·mL^−1^ for demethoxycurcumin, and 0.4699 to 1.958 *μ*g·mL^−1^ for bisdemethoxycurcumin.

### 2.7. Method Validation

The HPLC method was employed and methodology was examined for linearity, recovery, precision, repeatability, and stability. The validation was implemented based on the relative peak areas, the linear regression analysis was used to prepare calibration curves, and relative standard deviation (RSD) was used to evaluate precision, repeatability, stability, and recovery.

## 3. Results

### 3.1. Chromatographic Conditions

**Figure 1:**
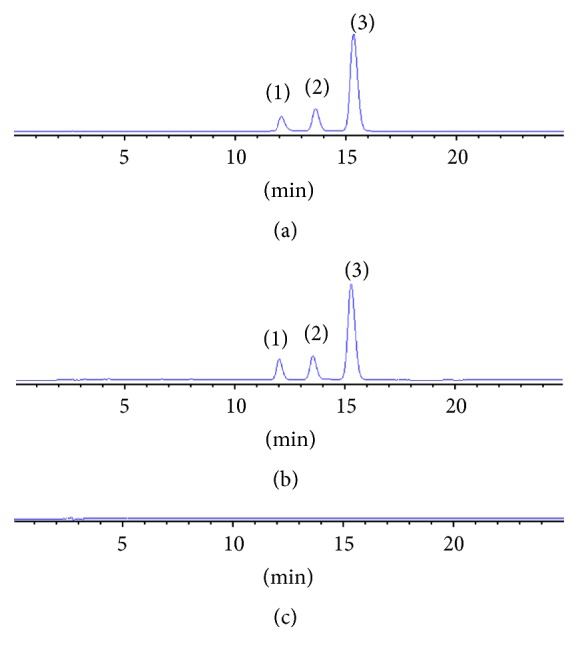
HPLC of reference substance (a),* Curcuma Longa Radix* sample (b), and negative control sample (c). (1) Bisdemethoxycurcumin, (2) demethoxycurcumin, and (3) curcumin.

Acetonitrile (A) and 4% glacial acetic acid aqueous (B) (V/V) were chosen as the composition of mobile phases for all the analyses. The chromatogram of mixed standard compounds, sample, and negative control sample were shown in Figure 1. In Figure 1, the three peaks marked with (1), (2), and (3) were bisdemethoxycurcumin, demethoxycurcumin, and curcumin. Mixed standard compounds and samples had the same retention time. The degrees of separation of curcumin, demethoxycurcumin, and bisdemethoxycurcumin were all greater than 1.5 and theoretical plate number was greater than 9000. The negative control sample had no peaks at the corresponding positions of* Curcuma Longa Radix* sample, which illustrated the other medicine herbs did not interfere with determination. The method gave good specificity.

### 3.2. Calibration Curves

Using the above chromatographic conditions, the calibration curves of 3 compounds exhibited good linear regressions.  Table 3 gave the linear ranges survey of contents of three curcuminoids.

### 3.3. Precision, Repeatability, Stability, and Recovery

The precision was obtained by six copies of determinations individually of the standard solution and their RSD were calculated. The repeatability was performed by six-time determinations continuously for a sample (S9). The stability was tested with the sample solution that was stored at room temperature at several time points (0, 2, 4, 8, 12, and 24 h after preparation), and the 3 compounds were found to be rather stable within 24 h (RSD < 3%). In the recovery test, 6 samples were prepared by spiking known quantity of each of the 3 standards into the* Curcuma Longa Radix* sample that had been measured and then extracted according to sample preparation and analyzed. All of these data were shown in Table 4.

### 3.4. Relative Correlation Factors (RCFs, *f*)

The mixed standard solution was injected 1 *μ*L, 2 *μ*L, 4 *μ*L, 6 *μ*L, 8 *μ*L, 10 *μ*L, and 20 *μ*L. Curcumin was used as the internal marker to calculate RCFs of demethoxycurcumin (A) and bisdemethoxycurcumin (B). The results were shown in Table 5.

*A*_*S*_ and *C*_*S*_ were, respectively, the peak area and concentration of internal marker, and *A*_*X*_ and *C*_*X*_ were, respectively, the peak area and concentration of analyte [15].

### 3.5. Effects of Various Factors on RCFs(*f*)

In this study, the effects of different instruments (Shimadzu LC-20A and Agilent 1200), chromatographic column (Welch Ultimate XB, SPOLAR, and Diamonsil), column temperature (25°C, 30°C, and 35°C), wavelength (423 nm, 425 nm, and 427 nm), and flow rate (0.90 mL/min, 0.95 mL/min, 1.00 mL/min, 1.05 mL/min, and 1.10 mL/min) on RCFs were investigated. All of these data were shown in Table 6.

### 3.6. Location of Chromatographic Peaks to Be Measured

The relative retention value (*r*_*X*/*S*_, which was the retention time ratio of analyte and internal marker) of* Curcuma Longa Radix* of each component under test was used for the location of the chromatographic peak. Curcumin was used as the internal marker to calculate *r*_*X*/*S*_ of demethoxycurcumin and bisdemethoxycurcumin. *r*_*X*/*S*_ of demethoxycurcumin and bisdemethoxycurcumin were 0.888 and 0.788 and RSD were 0.02% and 0.02%, respectively.

(1) rX/S=tRXtRSX was   analyte  and S was   the   internal   marker.

(see [16]).

### 3.7. Comparison between QAMS and External Standard Method (ESM)

Samples were prepared according to the preparation method of the sample solution, and the contents of curcumin, demethoxycurcumin, and bisdemethoxycurcumin in the samples were determined by HPLC. The contents of each component were calculated by using RCFs. Comparing results of QAMS and ESM, we find that the RSD were within 3%. So the QAMS used in the study of multicomponents quality evaluation of* Curcuma Longa Radix* was feasible. The results were shown in Table 7.

## 4. Discussion

In this paper, based on Chinese Pharmacopoeia 2015 edition Volume IV, preliminary experiments, and literature, we not only determined the homeopathic alcohol as the solvent and the extract of* Curcuma Longa Radix* were determined by hot dipping method but also increased the content of volatile oil of* Curcuma Longa Radix* on quality control.

The TCM theory believes that the efficacy of TCM is due to the multicompounds which consist of many different kinds of chemical constituents [17–21]. So it is difficult to accurately reflect the quality of traditional Chinese medicine by a single component as quality control indicators. In order to control the quality of traditional Chinese medicine, it is necessary to select a number of effective components or main components as indicator, especially the chemical components related to efficacy.

Curcumin, demethoxycurcumin, and bisdemethoxycurcumin are the major active components of* Curcuma Longa Radix*. Three components have clear chemical structure, obvious pharmaceutical properties, and convenient detection, so they should be considered for study first. QAMS, which has many advantages such as low cost and high efficiency, has been widely used in the determination of traditional Chinese medicine and multi-index components in recent years [17, 22–26]. In this experiment, the 3 components to be tested were curcumin derivatives and curcumin was a representative component of these compounds and is easy to obtain. So we established QAMS of* Curcuma Longa Radix* by using curcumin as the internal reference. The effects of different instruments, chromatographic column, column temperature, wavelength, and flow rate on RCFs were investigated. At the same time, 12 batches of* Curcuma Longa Radix* for verification were selected. The results showed that RCFs has good reproducibility under the experimental conditions and has no significant difference with ESM. Compared with ESM, QAMS overcomes the shortage of standard goods and saves testing fees, which makes it possible to apply to production practice.

## 5. Conclusions

TCM is the important part of Chinese culture. And how to establish an effective and reasonable method to monitor the quality of Chinese medicine is necessary. This study provides a new HPLC method for quality control of* Curcuma Longa Radix*. The results showed that the QAMS method for determination of curcumin derivatives of* Curcuma Longa Radix* was fast, accurate, and stable. The method could be suitable for quality control of* Curcuma Longa Radix*.

## Figures and Tables

**Table 1:** Information of *Curcuma Longa Radix* samples.

Number	Place of purchase	Time	Place of origin
(S1)	Co.A	20170104	Sichuan
(S2)	Co.B	20160824	Sichuan
(S3)	Co.B	20160728	Sichuan
(S4)	Co.B	20160310	Sichuan
(S5)	Zhoudu Village of Shuangliu	201602	Shuangliu (Sichuan)
(S6)	Zhoudu Village of Shuangliu	201510	Shuangliu (Sichuan)
(S7)	Zhoudu Village of Shuangliu	201701	Shuangliu (Sichuan)
(S8)	Sichuan Hehuachi medicine market	20170518	Sichuan
(S9)	Qianwei	20170705	Qianwei (Sichuan)
(S10)	Co.C	20161025	Sichuan
(S11)	Co.C	2017519	Sichuan
(S12)	Co.C	20170206	Sichuan

**Table 2:** Results of ethanol extracts and volatile oil (*n* = 3).

Number	Extracts (%)	Volatile oil (mL/100 g)
(S1)	14.602	0.55
(S2)	14.382	0.45
(S3)	13.509	0.75
(S4)	14.749	0.45
(S5)	15.644	0.70
(S6)	9.749	0.80
(S7)	10.109	0.50
(S8)	12.334	0.80
(S9)	14.987	0.90
(S10)	13.940	0.60
(S11)	13.671	0.75
(S12)	14.001	0.70

**Table 3:** Linear ranges survey of 3 curcuminoids in *Curcuma Longa Radix*.

Standard substance	Regression equation	Linear range (*μ*g/mL)	Correlation coefficient (*r*^2^)
Curcumin	*y* = 9096.4*x* − 20.331	3.3242~13.851	0.9997
Desmethoxycurumin	*y* = 9194.2*x* − 5.6895	0.6768~2.82	0.9997
Bisdesmethoxycurum	*y* = 8601.1*x* + 2.2162	0.4699~1.958	0.9993

**Table 4:** Precision, repeatability, stability, and recovery of 3 curcuminoids.

Compound	Precision	Repeatability	Stability	Recovery	
	RSD (%)	RSD (%)	RSD (%)	Mean	RSD (%)
Curcumin	0.38%	1.50%	0.74%	96.26%	2.58%
Desmethoxycurumin	0.40%	1.12%	0.83%	94.43%	2.47%
Bisdesmethoxycurum	0.39%	1.24%	0.95%	95.86%	3.97%

**Table 5:** *f*_*S*/*X*_^*∗*^ determination results with curcumin as internal content.

Injection volume/*μ*L	1	2	4	6	8	10	20	Mean	RSD%
*f*_*S*/A_	0.988	0.995	0.994	0.991	0.990	0.991	0.988	0.991	0.25
*f*_*S*/B_	1.080	1.068	1.060	1.054	1.050	1.090	1.045	1.064	1.54

^*∗*^ *f*_*S*/*X*_ = *f*_*S*_/*f*_*X*_ = (*A*_*S*_ × *C*_*X*_)/(*A*_*X*_ × *C*_*S*_).

**Table 6:** Effects of various factors on RCFs(*f*).

Effect	*f*_*S*/A_		*f*_*S*/B_	
	Mean	RSD (%)	Mean	RSD (%)
Instruments				
Shimadzu LC-20A	0.9788	1.02	1.0580	1.31
Agilent 1200	0.9931		1.0778	
HPLC column				
Welch Ultimate XB	0.9752	1.22	1.0398	0.8
SPOLAR	0.9993		1.0563	
Diamonsil	0.9886		1.0457	
Column temperature				
25°C	0.9903	0.06	1.0472	0.02
30°C	0.9899		1.0469	
35°C	0.9908		1.0467	
Wavelength				
423 nm	0.9798	0.96	1.0239	1.95
425 nm	0.9894		1.0445	
427 nm	0.9989		1.0645	
Flow Rate				
0.90 mL/min	0.9895	0.02	1.0460	0.06
0.95 mL/min	0.9892		1.0460	
1.00 mL/min	0.9889		1.0448	
1.05 mL/min	0.9889		1.0447	
1.10 mL/min	0.9892		1.0452	

**Table 7:** Results of sample determination by ESM and QAMS.

Number	Curcumin mg/g	Demethoxycurcumin mg/g		Bisdemethoxycurcumin mg/g	
		ESM	QAMS	ESM	QAMS
(S1)	0.919	0.153	0.152	0.078	0.080
(S2)	0.948	0.166	0.165	0.090	0.091
(S3)	1.040	0.167	0.167	0.088	0.089
(S4)	0.735	0.127	0.126	0.079	0.080
(S5)	1.369	0.241	0.239	0.144	0.145
(S6)	0.696	0.121	0.120	0.041	0.042
(S7)	0.104	0.029	0.030	0.037	0.040
(S8)	0.750	0.124	0.123	0.072	0.074
(S9)	0.766	0.160	0.160	0.137	0.139
(S10)	0.823	0.131	0.130	0.068	0.069
(S11)	1.087	0.160	0.159	0.069	0.070
(S12)	1.217	0.221	0.221	0.107	0.109

## References

[1] Chinese Pharmacopoeia (2015). *Chinese Pharmacopoeia Commission*.

[2] Tingmo Z. (2012). *Clinical Chinese pharmacy*.

[3] Zhou Y., Xie M., Song Y. (2016). Two traditional chinese medicines Curcumae Radix and Curcumae Rhizoma: an ethnopharmacology, phytochemistry, and pharmacology review. *Evidence-Based Complementary and Alternative Medicine*.

[4] Min L., ZHANG N., Qi-qu L. (2008). Study on quality control standard of Radix Curcuma Longa. *Journal of Chengdu University of TCM*.

[5] Shi X., Zheng Z., Li J. (2015). Curcumin inhibits A*β*-induced microglial inflammatory responses in vitro: involvement of ERK1/2 and p38 signaling pathways. *Neuroscience Letters*.

[6] Tohda C., Nakayama N., Hatanaka F., Komatsu K. (2006). Comparison of anti-inflammatory activities of six *Curcuma rhizomes*: a possible curcuminoid-independent pathway mediated by *Curcuma phaeocaulis* extract. *Evidence-Based Complementary and Alternative Medicine*.

[7] Oh S.-W., Cha J.-Y., Jung J.-E. (2011). Curcumin attenuates allergic airway inflammation and hyper-responsiveness in mice through NF-*κ*B inhibition. *Journal of Ethnopharmacology*.

[8] Yu D X., Cao H., Li J. (2014). Curcumin attenuates mechanical and thermal hyperalgesia in chronic constrictive injury model of neuropathic pain , pain therapy. *Curcumin Attenuates Mechanical and Thermal Hyperalgesia in Chronic Constrictive Injury Model of Neuropathic Pain , Pain Therapy*.

[9] Prasad S., Gupta S. C., Tyagi A. K., Aggarwal B. B. (2014). Curcumin, a component of golden spice: from bedside to bench and back. *Biotechnology Advances*.

[10] Liu L., Zhang P., Li Y., Yu G. (2012). Curcumin protects brain from oxidative stress through inducing expression of UCP2 in chronic cerebral hypoperfusion aging-rats. *Molecular Neurodegeneration*.

[11] Pyun C.-W., Kim J.-H., Han K.-H., Hong G.-E., Lee C.-H. (2014). *In vivo* protective effects of dietary *Curcumin* and capsaicin against alcohol-induced oxidative stress. *BioFactors*.

[12] Sarvalkar P. P., Walvekar M. V., Bhopale L. P. (2011). Antioxidative effect of curcumin (*Curcuma longa*) on lipid peroxidation and lipofuscinogenesis in submandibular gland of D-galactose- induced aging male mice. *Journal of Medicinal Plant Research*.

[13] Arora V., Kuhad A., Tiwari V., Chopra K. (2011). Curcumin ameliorates reserpine-induced pain-depression dyad: behavioural, biochemical, neurochemical and molecular evidences. *Psychoneuroendocrinology*.

[14] Li R., He X-y., Xiao Y. (2013). Quantitative analysis of curcuminoids in Huangsi Yujin and Lvsi Yujin derived from Cucurmae Radix. *Chinese Journal of Experimental Traditional Medical Formulae*.

[15] Xue-e Y., Jian-ping Q., Jia-chun L. (2017). Simultaneous determination of 7 flavonoid compounds in Yinyanghuo Zonghuangtong capsule by quantitative analysis of multi-components with a single-marker. *Chinese Journal of Experimental Traditiomal Medical Formulae*.

[16] Liu H-y., Shen G-b. (2017). Simultaneous determination of seven components in Chrysanthemum indicum by QAMS. *Chinese Traditional and Herbal Drugs*.

[17] Yang Y., Zhang G., Sun Q. (2017). Simultaneous determination of 8 compounds in Gancao-Ganjiang-Tang by HPLC-DAD and analysis of the relations between compatibility, dosage, and contents of medicines. *Evidence-Based Complementary and Alternative Medicine*.

[18] Ryu G., Weon J. B., Yang W. S., Ma C. J. (2017). Simultaneous determination of four compounds in a *Nelumbo nucifera* seed embryo by HPLC-DAD. *Journal of Spectroscopy*.

[19] Zhou Y., Wei W. L., Hua J. X., Fan Q. (2016). A simple and convenient method for simultaneous determination of Schizandrol A, Schizandrol B, Schisandrin A, *γ*-Schisandrin, and Schisandrin C. *Journal of Chemistry*.

[20] Huyiligeqi, Dong X., Yang C. (2016). Chemical constituents from daphne giraldii nitsche and their contents simultaneous determination by HPLC. *Evidence-Based Complementary and Alternative Medicine*.

[21] Li D.-H., Lv Y.-S., Liu J.-H. (2016). Simultaneous determination of four active ingredients in *Sargentodoxa cuneata* by HPLC coupled with evaporative light scattering detection. *International Journal of Analytical Chemistry*.

[22] Wen-bing L., Jun-rong L., Lin H. (2017). Determination of curcumin, desmethoxycurumin, and bisdesmethoxycurum in *Curcuma longa* by QAMS. *Chinese Traditional and Herbal Drugs*.

[23] Wen-jie W., Yang D., Gui-lin T. (2017). Determination of five isoflavonoids in *Pueraria Radix* by QAMS. *Chinese Traditional and Herbal Drugs*.

[24] Yu H., Tang L., Wu H. (2016). Determination of contents of four alkaloids in Pericarpium arecae by quantitative analysis of multi-components by single-marker. *Pakistan Journal of Pharmaceutical Sciences*.

[25] Yan C., Wu Y., Weng Z. (2015). Development of an HPLC method for absolute quantification and QAMS of flavonoids components in *Psoralea corylifolia* L. *Journal of Analytical Methods in Chemistry*.

[26] Jiang Y., Chen H., Wang L., Zou J., Zheng X., Liu Z. (2016). Quality evaluation of polar and active components in crude and processed fructus corni by quantitative analysis of multicomponents with single marker. *Journal of Analytical Methods in Chemistry*.

